# Comparison of two adult mosquito sampling methods with human landing catches in south-central Ethiopia

**DOI:** 10.1186/s12936-016-1668-9

**Published:** 2017-01-13

**Authors:** Oljira Kenea, Meshesha Balkew, Habte Tekie, Teshome Gebre-Michael, Wakgari Deressa, Eskindir Loha, Bernt Lindtjørn, Hans J. Overgaard

**Affiliations:** 1 Department of Zoological Sciences, Addis Ababa University, P. O. Box 1176, Addis Ababa, Ethiopia; 2 Department of Biology, Wollega University, P. O. Box 395, Nekemte, Ethiopia; 3 Akililu Lemma Institute of Pathobiology, Addis Ababa University, P. O. Box 1176, Addis Ababa, Ethiopia; 4 Department of Preventive Medicine, School of Public Health, College of Health Sciences, Addis Ababa University, P. O. Box 1176, Addis Ababa, Ethiopia; 5 School of Public and Environmental Health, Hawassa University, P. O. Box 1560, Hawassa, Ethiopia; 6 Centre for International Health, University of Bergen, P. O. Box 7804, 5020, Bergen, Norway; 7 Norwegian University of Life Sciences, P. O. Box 5003, 1432, Ås, Norway

**Keywords:** *Anopheles arabiensis*, Ethiopia, Human landing catches, Light traps

## Abstract

**Background:**

The human landing catch (HLC) is the standard reference method for measuring human exposure to mosquito bites. However, HLC is labour-intensive, exposes collectors to infectious mosquito bites and is subjected to collector bias. These necessitate local calibration and application of alternative methods. This study was undertaken to determine the relative sampling efficiency (RSE) of light traps with or without yeast-produced carbon dioxide bait vs. HLC in south-central Ethiopia.

**Methods:**

The experiment was conducted for 39 nights in a 3 × 3 Latin square randomized design with *Anopheles arabiensis* as the target species in the period between July and November 2014 in Edo Kontola village, south-central Ethiopia. Center for Disease Control and Prevention light trap catches (LTC) and yeast-generated carbon dioxide-baited light trap catches (CB-LTC) were each evaluated against HLC. The total nightly mosquito catches for each *Anopheles* species in either method was compared with HLC by Pearson correlation and simple linear regression analysis on log-transformed [log_10_(*x* + 1)] values. To test if the RSE of each alternative method was affected by mosquito density, the ratio of the number of mosquitoes in each method to the number of mosquitoes in HLC was plotted against the average mosquito abundance.

**Results:**

Overall, 7606 *Anopheles* females were collected by the three sampling methods. Among these 5228 (68.7%) were *Anopheles ziemanni*, 1153 (15.2%) *An. arabiensis*, 883 (11.6%) *Anopheles funestus* s.l., and 342 (4.5%) *Anopheles pharoensis*. HLC yielded 3392 (44.6%), CB-LTC 2150 (28.3%), and LTC 2064 (27.1%) *Anopheles* females. The RSEs of LTC and HLC for *An. arabiensis* were significantly correlated (p < 0.001) and density independent (p = 0.65). However, for outdoor collection of the same species, RSEs of LTC and CB-LTC were density dependent (p < 0.001). It was estimated that on average, indoor LTC and CB-LTC each caught 0.35 and 0.44 times that of indoor HLC for *An. arabiensis* respectively.

**Conclusions:**

Results showed that HLC was the most efficient method for sampling *An. arabiensis*. LTC can be used for large-scale indoor *An. arabiensis* surveillance and monitoring when it is difficult to use HLC. CB-LTC does not substantially improve sampling of this major vector compared to LTC in this setting.

*Trial registration* PACTR201411000882128 (retrospectively registered 8 September, 2014)

## Background

The current core malaria vector interventions are long-lasting insecticidal nets (LLINs) and indoor residual spraying (IRS) [1]. In attempts to control malaria by attacking the vector, it is important to measure the impact of such interventions on mosquito populations. Measuring this requires an appropriate method of sampling mosquitoes biting humans [2, 3]. The most direct way to do this is by the human landing catch (HLC) because mosquitoes are captured as they land and attempt to feed on collectors [4]. The HLC is the standard method for measuring exposure of humans to mosquito bites [5] and for estimating the human biting rate (HBR) which is a key determinant of the entomological inoculation rate (EIR), a measure of malaria transmission [6].

However, the HLC exposes collectors to potentially infectious mosquito bites, is labour-intensive, requires highly trained collectors and is difficult to supervise. Besides, results obtained by HLC can be biased due to natural human variations in attractiveness to mosquitoes [7, 8]. These issues limit the application of HLC particularly for monitoring the effectiveness of vector control interventions and necessitate the search for alternative methods.

Several mosquito sampling methods that do not require human exposure have been developed as alternative to HLC for estimating the HBR. For African malaria vectors, the main alternative mosquito sampling methods include the standard Centers for Disease Control and Prevention light trap catches (LTC) placed beside human-occupied bed nets [2, 4, 9–11], bed net traps [12, 13], tent traps [3, 14–17], odour-baited traps [18, 19] and electrocution traps [20, 21].

A recent review by Briet et al. [8] showed that LTC has been widely evaluated against HLC for collecting host-seeking vectors in several areas and is considered a safe and approximately equivalent alternative to HLC for measuring indoor exposure to mosquito bites and malaria transmission by African vectors. Light traps are affordable, easy to use and can be deployed in large-scale longitudinal, community-based trapping schemes using solar-powered battery chargers and provide valuable entomological data of the impact of vector control interventions [16, 22].

Another promising alternative to HLC is carbon dioxide-baited traps. Many kairomones are released by the human body, but carbon dioxide (CO_2_) is the most important for luring host-seeking mosquitoes [23]. Artificial sources of CO_2_, specifically from dry ice, industrial pressurized gas cylinders, or from propane are commonly used in mosquito traps [22, 23]. However, adult mosquito surveillance in many rural areas is challenging due to lack of CO_2_, either in the form of dry ice or compressed gas. In resource-poor areas, like sub-Saharan Africa, it is hard to obtain CO_2_ sources that are reliable, cheap, and durable [23].

In Japan, Saitoh et al. [24] developed an easy and cheap method to produce CO_2_ by using a yeast-sugar solution in plastic bottles and evaluated the efficacy of yeast-generated CO_2_ as an attractant for mosquitoes in field collections. They found that traps baited with yeast-generated CO_2_ caught higher numbers of mosquitoes than unbaited traps. Results from trapping experiments conducted in the laboratory and semi-field systems in Kenya found that traps baited with yeast-produced CO_2_ caught significantly more *Anopheles gambiae* sensu stricto than unbaited traps as well as traps baited with industrial CO_2_ [23]. However, studies on other major African malaria vectors, such as *An. arabiensis* as a target species is lacking.

*Anopheles arabiensis* is the principal malaria vector in Ethiopia [25] and its control is primarily based on IRS and LLINs either in combination or separately. To generate evidence on the effect of IRS and LLIN combined interventions, a cluster randomized controlled trial was implemented in Adami Tullu district in south-central Ethiopia [26]. For this trial, an appropriate mosquito sampling method was required to monitor the impact of the interventions on *An. arabiensis* and other local *Anopheles* populations.

Although, CDC light traps have been used to estimate human biting rates and EIR equivalent to the ones obtained from HLC by locally calibrating against HLC and calculating conversion factors [2, 27], no such evaluation and determination of conversion factors have been carried out in Ethiopia so far. For example, Animut et al. [28] estimated daily EIR for *An. arabiensis* in highland areas of south-central Ethiopia based on a conversion factor for LTC vs. HLC of 1.91, determined for this species in Zambia [27]. Likewise, Massebo et al. [29] estimated annual EIR for the same species in Chano, south-western Ethiopia, using 1.605 a factor determined in Tanzania [2]. The efficiency of a collection method can vary according to the composition of the mosquito species present, mosquito densities, availability of alternative hosts, and city lighting [8], therefore, it is difficult to extrapolate a conversion factor from one local epidemiological situation to another. Thus, as part of the trial, this study was undertaken to evaluate the collection efficacy of light traps, carbon dioxide baited light traps vs. HLC. The objective was to assess which method could be used in place of HLC for routine mosquito surveillance by determining conversion factors between LTC and HLC and between CB-LTC and HLC.

## Methods

### The study area

**Fig. 1:**
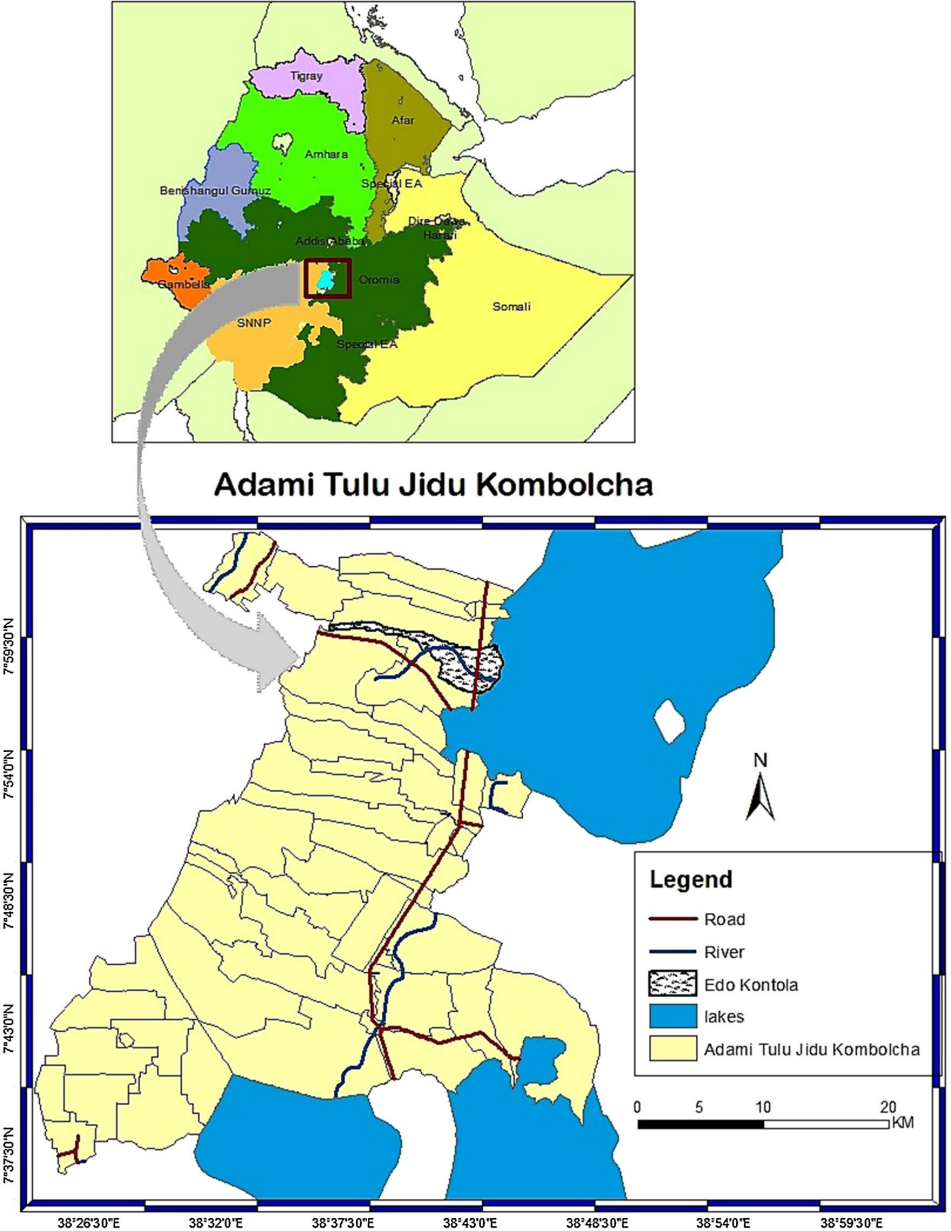
Map of Edo Kontola, in Adami Tullu district and its location in Ethiopia

The study area has been described in detail elsewhere [25, 26, 30]. In brief, the study was conducted in Edo Kontola, in Adami Tullu district, central Ethiopia (Fig. 1) during July–November 2014. This time coincides with the major malaria transmission which usually is from September to November. This village was selected based on results from preliminary mosquito collections showing high numbers of mosquitoes compared to other sites [31]. The capital of the district, Batu (formerly Zeway) is located at 7°56′N 38°42′E; 1640 meters above sea level. It is about 160 km south of Addis Ababa on the highway connecting Addis Ababa to Nairobi. The total annual rainfall was about 700 mm in 2013, with peaks in July (250 mm) and August (220 mm). The mean minimum and maximum annual temperatures were 14.5 and 27.7 °C, respectively. Most of the population in the district is living in rural areas in houses made of mud or cement walls and thatched or corrugated iron roofs. Local residents primarily depend on farming, livestock rearing, and fishing for subsistence from Lake Zeway [26].

### Study design and mosquito collections

**Fig. 2:**
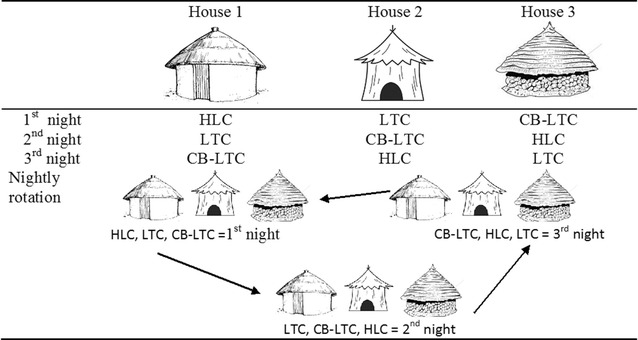
A 3 × 3 Latin square randomized design and rotational design for the three mosquito sampling methods for one round cycle of *Anopheles* collection in Edo Kontola, Ethiopia 2014

This mosquito sampling method calibration study was conducted simultaneously with monitoring of human-biting activities of local *Anopheles* species by HLC alone [30]. The experiment was conducted for 39 nights in a 3 × 3 Latin square randomized design replicated 13 times (cycles) in the period between July and November 2014 (Fig. 2). Three mosquito sampling methods were used:(1) Human landing catches (HLC), (2) CDC light trap beside a human-occupied bed net (LTC) and (3) CDC light trap baited with yeast-generated CO_2_ (CB-LTC). Eight local volunteers were trained on how to collect mosquitoes. Three houses of approximately similar size and design were selected. The houses were of traditional style with thatched conical-shaped roofs, circular floors and plastered walls. It was also arranged in such a way that the selected houses were free of cattle, chicken and human occupants on all collection nights.

On each experimental night, four of the eight volunteers were allocated to one of the three houses to perform HLC, while the other four were assigned to sleep next to the traps individually under LLINs in indoor and outdoor venues of the other two houses. The sampling methods were rotated among the houses nightly. Mosquito collections were mainly done for three consecutive nights per week. The traps operated overnight from 19:00 to 06:00 h. Human landing catches were performed for 50 min each hour with 10 min break for the collectors. In a house assigned to HLC, four collectors conducted HLC in two rounds on each experimental night. During the first round, from 19:00 to 24:00 h, one person collected mosquitoes indoors and the other outdoors. From 24:00 to 06:00 h, the other collectors took over and performed the same activities. Collectors sat on chairs indoors and outdoors with their legs exposed; the outdoor collector was positioned at least 10 m from the house. Using flashlights, collectors caught landing mosquitoes with a hand-held mouth aspirator and each hour’s collection was kept separately in labelled paper cups.

Indoor and outdoor collectors changed venues during the 10 min break whereas the two groups of collectors changed for pre- and post-midnight shifts alternately each night, i.e. the group that collected during pre-midnight hours worked during the post-midnight period the next night and vice versa. The collectors worked during different times and sites to reduce the effects of a particular site and compensate individual differences in attractiveness of the human baits. At a house assigned to CDC light trap alone, two light traps, one indoor and the other outdoor, were hung at the feet of sleeping volunteers, who were protected by a LLIN. Traps were generally positioned at 1.0 m from the floor or ground, where the outdoor light trap was set 10 m from the outer wall of the house and on the opposite wall from where the indoor light trap was placed.

Two plastic bottles of each 2.5 l volume were used to hold yeast-sugar solution for fermentation and production of CO_2_ to be used in CB-LTC. This was made by mixing 17.5 g dry bakers’ yeast and 250 g table sugar in 250 ml tap water [24] at ambient temperature 1 h before the set up and operation of the traps on each experimental night. Silicon tubes each with 0.70 mm diameter were fitted through a hole drilled in the screw cap to release CO_2_ to the vicinity of the light bulb of the trap. At a house assigned to this collection method, a CB-LTC was set indoors at the feet of a sleeping volunteer who was protected by LLIN similar to LTC as described above. The same procedure was applied for the outdoor CB-LTC, which was placed at 10 m distance from the outer wall of the house.

### Mosquito processing

Live mosquitoes captured by the above methods were killed with chloroform. At a field laboratory in Batu town, mosquitoes were first sorted out to Culicines and Anophelines. Adult female *Anopheles* mosquitoes were further identified by morphological criteria using identification keys [32]. *An. arabiensis* was confirmed to be the only member of *An. gambiae* species complex in the study area [25, 31] and also in nearby areas [28, 29]. In order to determine malaria sporozoite infection rate, the head and thorax of each mosquito (n = 6295) were carefully separated from the abdomen and tested for the presence of *Plasmodium falciparum* and *Plasmodium vivax* circumsporozoite proteins (CSP) by the direct enzyme-linked immunosorbent assay (ELISA) [33].

### Ethical considerations

The ethical considerations for this study is described in more details in the published protocol [26] and is specifically outlined as follows:

#### Ethical approval

Ethical approval for the study was obtained from the Institutional Review Board of the College of Health Sciences at Addis Ababa University, the Ministry of Science and Technology in Ethiopia (Ref: 3.10/446/06), and the Regional Committee for Medical and Health Research Ethics, (Ref: 2013/986/REK Vest) Western Norway [26]. The protocol for the trial was registered at the Pan African Clinical Trials Registry under the Registration Number PACTR201411000882128.

### Information and informed consent

Verbal and written informed consent to take part in the study was obtained beforehand from volunteers for landing catches, who were all more than 18 years old and from house owners using the local Afan Oromo language. For the human landing catches, a separate written informed consent describing the potential risks and benefits of the study was obtained from the volunteers. These volunteers were selected from the study village. The participants were instructed that involvement in the study was voluntary, and that they had the right to withdraw at any time regardless of reason. Assurance was also given that a refusal to participate in this study would not affect their access to services at the health posts in the study villages in the community.

### Malaria treatment

Mosquito collectors were trained to collect mosquitoes as soon mosquitoes land and before they bite. To help minimize risk, data collectors for the human landing catches were provided with an appropriate prophylactic drug (Malarone) before the collections. There were no reports on Malarone resistant *Plasmodium* parasites in Ethiopia. The project provided blood examination and treatment of malaria free of charge for any study participant or householder who fell ill or wished to check himself. The project follows the examination and treatment guidelines as described in the study protocol [26]. Fortunately, none of the mosquito collectors or householders was found parasite-positive during the study period.

### Data analysis

Data entry and analyses were performed using the SPSS version 20.0. Data from the HLC were divided by 0.83, i.e. 50/60, to account for the fact that HLC was performed for only 50 min of each hour. The nightly number of mosquitoes (*x*) caught by each method was transformed to log10 (*x* + 1), to normalize the distribution. Differences among the sampling methods, collection venues (indoor/outdoor), dates of collection and mosquito species were evaluated by analysis of variance (ANOVA) and Tukey’s Post-hoc test. To determine whether each of the alternative sampling method were correlated with the reference method (HLC), the Pearson correlation coefficients for the relationship among log-transformed catches for each *Anopheles* species was analyzed. The nightly mosquito catches for each *Anopheles* species in each alternative method were compared with those of the HLC by a simple linear regression analysis on log-transformed values [34].

The relative sampling efficiency (RSE) was measured as the ratio of the number of mosquito species caught by each alternative method to the number caught by the reference method [34]. To test if the RSEs of LTC and CB-LTC were each affected by mosquito density, the ratios of the numbers of mosquitoes in each alternative method to the number of mosquitoes in HLC [log(HLC + 1)−log(LTC + 1)], was plotted against the average mosquito abundance, calculated as [log(HLC + 1) + log(LTC + 1)]/2 [34]. Results were considered significant at p < 0.05. Mean log ratio and its antilog (geometric mean ratio) was used to estimate conversion factors between each of the alternative traps (LTC and CB-LTC) and the reference method (HLC) for *Anopheles* species that showed consistent relative sampling efficiencies, i.e. that were not dependent on mosquito density [2].

## Results

### *Anopheles* abundance and density

Overall, 7606 adult *Anopheles* females were collected by the three sampling methods over the 39 trap nights (Table 1). Among these 5228 (68.7%) were *Anopheles ziemanni*, 1153 (15.2%) *Anopheles arabiensis*, 883 (11.6%) *Anopheles funestus* s.l. and 342 (4.5%) *Anopheles pharoensis*. HLC captured the highest number of anophelines, 3392 (44.6%), followed by CB-LTC 2150 (28.3%) and LTC 2064 (27.1%). Similarly, indoor catches by HLC, LTC and CB-LTC were 766 (35.2%), 726 (33.3%) and 685 (31.5%) respectively. The corresponding outdoor catches were 2626 (48.4%) by HLC, 1465 (27%) by CB-LTC and 1338 (24.6%) by LTC.

**Table 1:** Number and proportions of *Anopheles* species collected indoors (IN) and outdoors (OUT) by three collection methods in Edo Kontola, Ethiopia 2014

*Anopheles*	Venue	HLC		LTC		CB-LTC		Sum	
		n	%	n	%	n	%	n	%
*An. arabiensis*	IN	370	57.7	123	19.2	148	23.1	641	
	OUT	463	90.4	17	3.3	32	6.3	512	
	Total	833	72.2	140	12.1	180	15.7	1153	15.2
*An. pharoensis*	IN	44	31.6	60	43.2	35	25.2	139	
	OUT	180	88.7	14	6.9	9	4.4	203	
	Total	224	65.5	74	21.6	44	12.9	342	4.5
*An. ziemanni*	IN	330	36.9	323	36.1	241	26.9	894	
	OUT	1945	44.9	1111	25.6	1278	29.5	4334	
	Total	2275	43.5	1434	27.4	1519	29.1	5228	68.7
*An. funestus* s.l.	IN	22	4.4	220	43.7	261	51.9	503	
	OUT	38	10.0	196	51.6	146	38.4	380	
	Total	60	6.8	416	47.1	407	46.1	883	11.6
Total	IN	766	35.2	726	33.3	685	31.5	2177	28.6
	OUT	2626	48.4	1338	24.6	1465	27.0	5429	71.4
Overall *Anopheles*		3392	44.6	2064	27.1	2150	28.3	7606	100.0

*HLC* human landing catch, *LTC* light trap catch and CB-LTC:CO_2_ baited light trap catch

*Anopheles arabiensis* was most abundant in HLC (n = 833, 72.2%) and least in LTC (n = 140, 12.1%). Conversely, *Anopheles funestus* s.l. was most abundant in LTC (n = 416, 47.1%) and least in HLC (n = 60, 6.8%). All the *Anopheles* species were most frequent in HLC except *Anopheles funestus* s.l. All species obtained by HLC were also collected by the other methods. Out of 234 mosquito sampling occasions over 39 nights (indoor and outdoor LTC, CB-LTC and HLC combined), there were five (2.1%) occasions without any *Anopheles* mosquitoes collected. Of the five zero catches, two occurred in outdoor CB-LTC, and one each in indoor LTC, outdoor LTC, and indoor CB-LTC, respectively. No zero catches occurred in indoor and outdoor HLC.

**Fig. 3:**
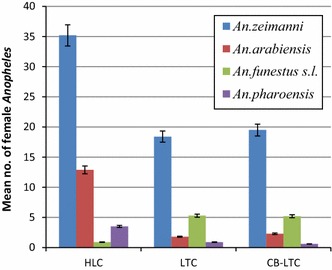
Mean number of female *Anopheles* species collected per man per night by HLC and per trap per night by LTC and CB-LTC (*Error bars* represent 95% confidence interval)

The mean *Anopheles* mosquito catches per trap night for each species is given in Fig. 3. The average density of female *Anopheles* collected by HLC was 52.4 (95% CI 39.9–66.2) mosquitoes per man per night and the corresponding values of CB-LTC and LTC were 27.6 (95% CI 18.4–37.6) and 26.5 (95% CI 17.6–35.6) mosquitoes per trap per night, respectively.

There were statistically significant differences among the average number of *Anopheles* species captured by HLC (F = 38.12, df = 3, p = 0.001), LTC (F = 11.17 df = 3, p = 0.001) and CB-LTC (F = 14.04, df = 3, p = 0.001). Post-hoc analyses showed that HLC yielded significantly higher mean numbers of *An. ziemanni* (F = 5.23, df = 2, p < 0.05), *An. arabiensis* (F = 60.14, df = 2, p < 0.001) and *An. pharoensis* (F = 36.26, df = 2, p < 0.001) compared to either of the alternative methods. However, HLC caught significantly lower numbers of *Anopheles funestus s.l.* than either LTC or CB-LTC (F = 16.33, df = 2, p = 0.001). Likewise, average mosquito catches by the three methods significantly varied between collection venues (F = 14.98, df = 5, p = 0.001). However, the average mosquito catches per house by HLC (F = 0.417, df = 38, p > 0.05), LTC (F = 1.037, df = 38, p > 0.05) and CB-LTC (F = 1.23, df = 38, p > 0.05) did not vary significantly by date of collection.

### Relative sampling efficiency of the alternative traps versus human landing catch

#### *Anopheles arabiensis*

**Fig. 4:**
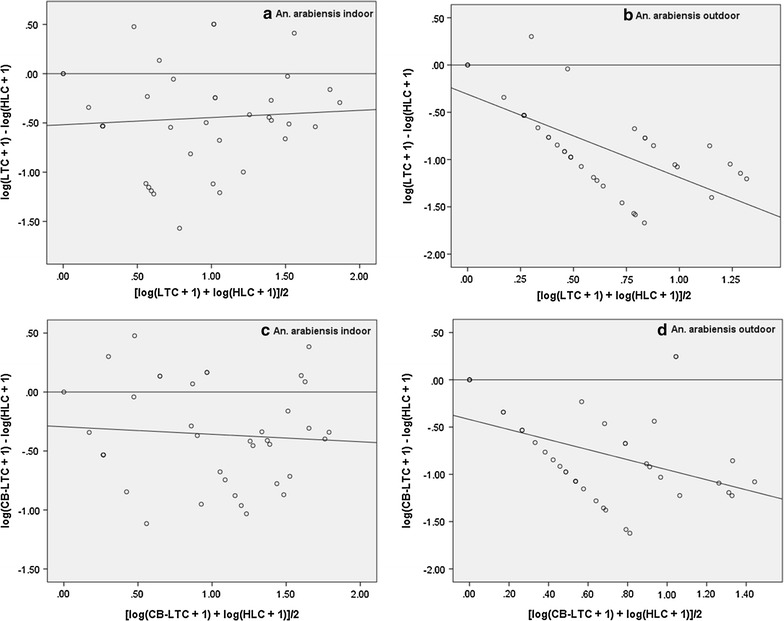
Relationship between relative sampling efficiency of indoor (**a**) and outdoor (**b**) LTC (*upper panels*), indoor (**c**) and outdoor (**d**) CB-LTC (*lower panels*) and abundance of *An. arabiensis*. Relative sampling efficiency is the difference in the mosquito catches by either of the alternative methods and the human landing catch (*y*-*axis*). The mosquito abundance is the joint average of each alternative and the reference method (*x*-*axis*)

There was a weak positive correlation between indoor LTC and HLC for this species (r = 0.31) and the regression slope was not significantly different from zero (Table 2; Fig. 4a), which means that the RSE of the indoor light traps were not dependent on mosquito density. The correlation between LTC and HLC for outdoor catches was positive (r = 0.38) and significant, but the RSE of light traps was significantly dependent on outdoor abundance (Table 2; Fig. 4b). Significant positive correlation (r = 0.49) was found between indoor CB-LTC and HLC; the RSE was not significantly dependent on mosquito density (Table 2; Fig. 4c). However, for the outdoor CB-LTC and HLC, the regression slope was significantly different from zero (Table 2; Fig. 4d) meaning that RSE of outdoor CB-LTC was dependent on mosquito density.

**Table 2:** Correlation and regression analysis of log-transformed indoor (IN) and outdoor (OUT) human landing catches (HLC) with either light trap (LTC) or yeast-generated CO_2_-baited light trap catches (CB-LTC) of *Anopheles* species in Edo Kontola, Ethiopia 2014

Species	Alternative vs. HLC	Venue	Correlation coefficient			Regression slope			
			n	r	p	b	95% CI	t	p
*An. arabiensis*	LTC	IN	39	0.308	0.056	0.073	−0.25 to 0.40	0.451	0.654
		OUT	39	0.378	<0.05	−0.880	−1.20 to 0.56	−5.565	<0.001
	CB-LTC	IN	39	0.493	0.001	−0.063	−0.33 to 0.20	−0.471	0.640
		OUT	39	0.288	0.076	−0.691	−0.91 to 0.47	−6.393	<0.001
*An. pharoensis*	LTC	IN	39	−0.019	0.906	0.519	0.14 to 0.89	2.792	<0.05
		OUT	39	0.243	0.136	−0.352	−0.73 to 0.03	−1.839	0.074
	CB-LTC	IN	39	0.235	0.150	0.078	−0.27 to 0.43	0.443	0.660
		OUT	39	0.133	0.419	−0.622	−1.05 to 0.18	−2.897	<0.05
*An. ziemanni*	LTC	IN	39	0.427	<0.05	0.081	−0.20 to 0.36	0.581	0.565
		OUT	39	0.775	<0.001	0.034	−0.12 to 0.19	0.442	0.661
	CB-LTC	IN	39	0.627	<0.001	0.005	−0.21 to 0.22	0.048	0.962
		OUT	39	0.795	<0.001	0.109	−0.03 to 0.25	1.544	0.131
*An. funestus s.l.*	LTC	IN	39	−0.164	0.317	0.948	0.71 to 1.18	8.084	<0.001
		OUT	39	0.316	0.050	0.522	0.28 to 0.75	4.488	<0.001
	CB-LTC	IN	39	0.024	0.885	0.846	0.63 to 1.05	8.220	<0.001
		OUT	39	0.463	<0.05	0.423	0.21 to 0.63	4.051	<0.001

The correlation coefficients show the relationship between log(LTC + 1) and log(HLC + 1), log(CB-LTC + 1) and log(HLC + 1). The regression slopes are from regressing relative sampling efficiencies (log(LTC + 1) – log (HLC + 1)) on average abundance ([log (LTC + 1) + log(HLC + 1)]/2) and also (log (CB-LTC + 1) − log (HLC + 1)) on average abundance ([log(CB-LTC + 1) + log(HLC + 1)]/2)

*n* sample size, *r* Pearson’s correlation coefficient, *b* regression slope, *CI* confidence interval, *t t* test value, *p* probability value

#### *Anopheles pharoensis*

**Fig. 5:**
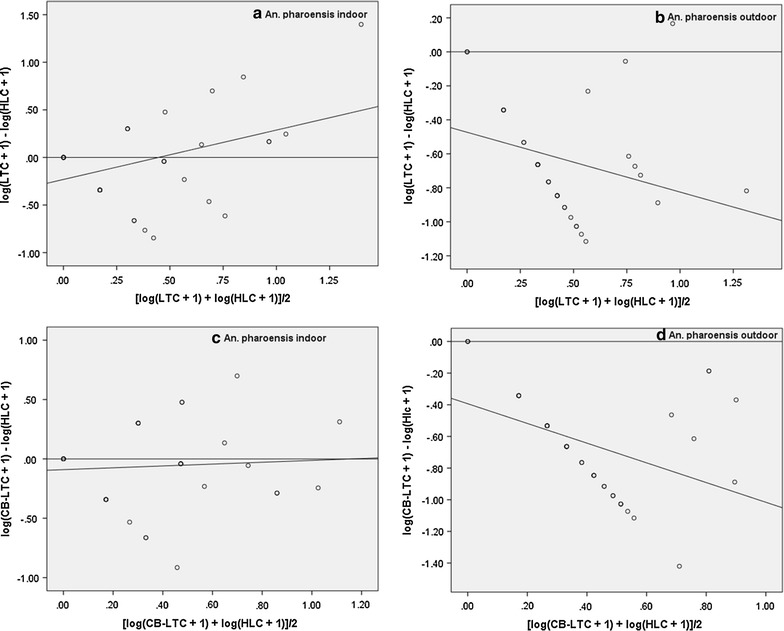
Relationship between relative sampling efficiency of indoor (**a**) and outdoor (**b**) LTC (*upper panels*), indoor (**c**) and outdoor (**d**) CB-LTC (*lower panels*) and density of *An. pharoensis*. Relative sampling efficiency is the difference in the mosquito catches by either of the alternative methods and the human landing catch (*y*-*axis*). The mosquito abundance is the joint average of each alternative and the reference method (*x*-*axis*)

For this species, there were no significant correlations between LTC and HLC indoors or outdoors nor between CB-LTC and HLC indoors and outdoors (Table 2). The RSEs of indoor LTC and outdoor CB-LTC compared to HLC, respectively, were significantly dependent on mosquito density (Table 2; Fig. 5a, d). However, the RSEs of outdoor LTC and indoor CB-LTC compared to HLC, respectively did not depend on mosquito density (Table 2; Fig. 5b, c).

#### *Anopheles ziemanni*

**Fig. 6:**
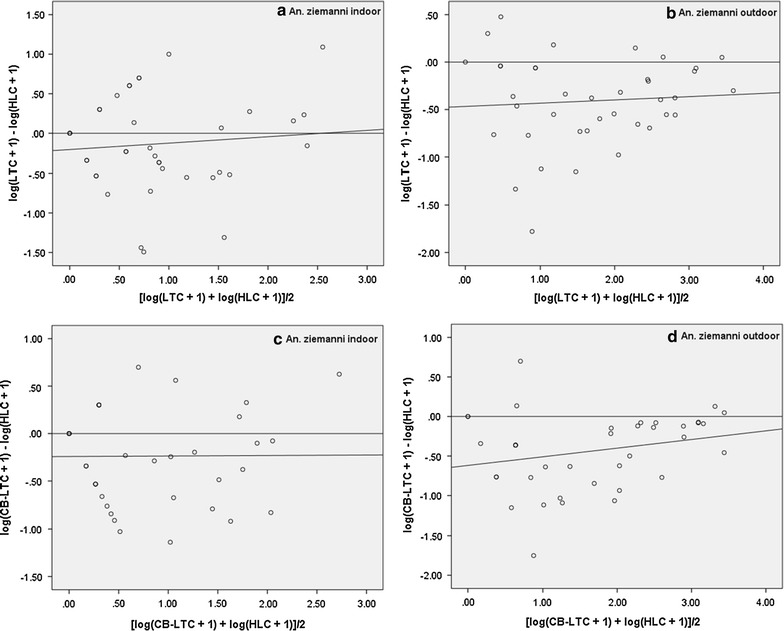
Relationship between relative sampling efficiency of indoor (**a**) and outdoor (**b**) LTC (*upper panels*), indoor (**c**) and outdoor (D) CB-LTC (*lower panels*) and abundance of *An. ziemanni*. Relative sampling efficiency is the difference in the mosquito catches by either of the alternative methods and the HLC (*y*-*axis*). The mosquito abundance is the joint average of each alternative and the reference method (*x*-*axis*)

A positive significant correlation was found between LTC and HLC indoors (r = 0.43) as well as outdoors (r = 0.78). Similarly, there were positive and significant correlations between CB-LTC and HLC both indoors (r = 0.63) and outdoors (r = 0.80). None of the regression slopes were significantly different from zero (Table 2; Fig. 6a–d), meaning that the RSEs were not dependent on mosquito density.

#### *Anopheles funestus* s.l

**Fig. 7:**
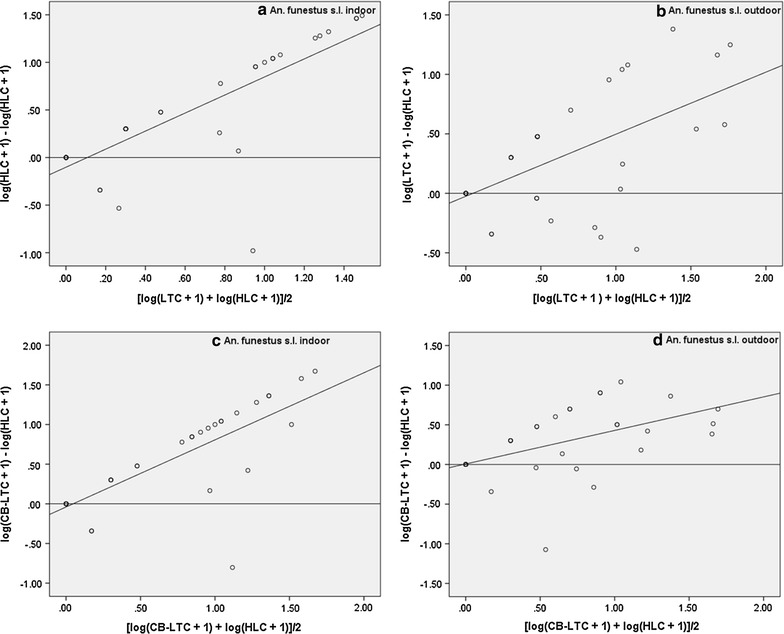
Relationship between relative sampling efficiency of indoor (**a**) and outdoor (**b**) LTC (*upper panels*), indoor (**c**) and outdoor (**d**) CB-LTC (*lower panels*) and abundance of *An. funestus* s.l.. Relative sampling efficiency is the difference in the mosquito catches by either of the alternative methods and the HLC (*y*-*axis*). The mosquito abundance is the joint average of each alternative and the reference method (*x*-*axis*)

For this species complex, all the regression slopes were significantly different from zero, indicating that both LTC: HLC ratio and CB-LTC:HLC ratio were dependent on the mosquito density indoors and outdoors. That means both of the alternative traps were not consistent for sampling *An. funestus* s.l. (Table 2; Fig. 7a–d).

### Conversion factors

The mean log ratios of LTC and CB-LTC vs. HLC, respectively were negative (Table 3), meaning that the RSEs were lower than HLC and thus less efficient than HLC for mosquito sampling in this setting. On average, the indoor LTC was 0.35 times that of indoor HLC and the indoor CB-LTC was 0.44 times that from HLC for *An. arabiensis*. For *An. pharoensis*, outdoor LTC caught on average 0.24 times that of outdoor HLC while indoor CB-LTC captured 0.86 times indoor HLC. However, for *An. ziemanni* on average, the catches from indoor and outdoor LTC were 0.73 and 0.39 times that from indoor and outdoor HLC, respectively. For the same species, on average indoor and outdoor CB-LTC captured 0.58 and 0.36 times the mosquito catch size of that of indoor and outdoor HLC, respectively.

**Table 3:** Mean log ratios and corresponding geometric mean ratios of alternative mosquito collection methods (light traps, LTC and CO_2_-baited light traps, CB-LTC) against the reference method (human landing catches, HLC) for sampling *Anopheles* species in Edo Kontola, Ethiopia

Species	Alternative method vs. HLC	Venue	Mean log ratio^a^	Standard error of the mean (S. E. M.)	Geometric mean ratio (GMR)	95% CI
*An. arabiensis*	LTC	IN	−0.4528	0.081	0.35	0.24–0.50
	CB-LTC	IN	−0.3578	0.067	0.44	0.33–0.59
*An. pharoensis*	LTC	OUT	−0.6281	0.052	0.24	0.18–0.29
	CB-LTC	IN	−0.0640	0.052	0.86	0.67–0.92
*An. ziemanni*	LTC	IN	−0.1341	0.095	0.73	0.48–0.87
	LTC	OUT	−0.4098	0.074	0.39	0.27–0.54
	CB-LTC	IN	−0.2379	0.076	0.58	0.41–0.81
	CB-LTC	OUT	−0.4389	0.075	0.36	0.25–0.49

^a^Negative mean log ratio indicates that the efficiency of LTC or CB_LTC were lower than HLC

### Relative sampling efficiency of the light trap with versus without CO_2_ bait

Relative sampling efficiency of LTC versus CB-LTC is given in Table 4. For *An. arabiensis*, there was a weak positive (r = 0.31) correlation between LTC and CB-LTC indoors but there was no significant correlation outdoors. The regression slopes were neither significantly different from zero indoors nor outdoors meaning that the RSEs were not dependent on indoor and outdoor *An. arabiensis* density in this setting. For *An. pharoensis*, the correlations between LTC and CB-LTC, both indoors and outdoors, were not significant. The RSEs of LTC and CB-LTC were significantly dependent on mosquito density both indoors and outdoors for this species. For *An. ziemanni*, a positive significant correlation was observed between LTC and CB-LTC indoors as well as outdoors and the RSEs were not dependent on mosquito density. Similarly, there were significant positive correlations between LTC and CB-LTC indoors and outdoors and the relation was not dependent on mosquito abundance indoors and outdoors for *An. funestus* s.l.

**Table 4:** Correlation and regression analysis of log-transformed indoor and outdoor light trap catch (LTC) with yeast-generated Co_2_-baited light trap catches (CB-LTC) of *Anopheles* species in Edo Kontola, Ethiopia 2014

Species	Venue	Correlation coefficient			Regression slope			
		n	r	p	b	95% CI	t	p
*An. arabiensis*	IN	39	0.311	0.054	0.287	−0.01 to 0.57	1.992	0.054
	OUT	39	−0.073	0.659	0.059	−0.44 to 0.56	0.237	0.814
*An. pharoensis*	IN	39	0.177	0.281	0.524	0.23 to 0.81	3.711	0.001
	OUT	39	−0.105	0.526	0.666	0.29 to 1.03	3.631	0.001
*An. ziemanni*	IN	39	0.643	<0.001	0.105	−0.09 to 0.31	1.056	0.298
	OUT	39	0.852	<0.001	−0.046	−0.17 to 0.08	−0.727	0.472
*An. funestus* s.l.	IN	39	0.525	0.001	0.082	−0.16 to 0.32	0.679	0.501
	OUT	39	0.507	0.001	0.179	−0.06 to 0.41	1.514	0.138

The correlation coefficients show the relationship between log(LTC + 1) and log(CB-LTC + 1). The regression slopes are from regressing relative sampling efficiencies (log(CB-LTC + 1)−log(LTC + 1)) on average abundance ([log(CB-LTC + 1) + log(LTC + 1)]/2)

*n* sample size, *r* Pearson’s correlation coefficient, *b* regression slope, *CI* confidence interval, *t t*-test value, *p* probability value

### Malaria infection

A total of 4000 *An. ziemanni*, 1153 *An. arabiensis*, 342 *An. pharoensis* and 800 *An. funestus* s.l. were tested for the presence of CSP of *P. falciparum* and *P. vivax*. However, none was found positive. For this reason, the entomological inoculation rate (EIR) could not be determined and compared among the sampling methods.

## Discussion

The ultimate aim of this study was to determine reliable conversion factors between either light trap (LTC) alone or light traps baited with yeast-generated carbon dioxide (CB-LTC) both set beside occupied long-lasting insecticidal nets against human landing (HLC) for entomological monitoring of the impact of malaria control interventions. The results showed that the HLC was the most efficient method compared to both LTC and CB-LTC for sampling the majority of *Anopheles* species including the major malaria vector, *An. arabiensis*. Despite lower relative *Anopheles* sampling efficiencies of both LTC and CB-LTC compared to HLC, they can be used as alternative to indoor HLC of *An. arabiensis*.

It was estimated that on average, indoor LTC caught 0.35 times the number of *An. arabiensis* as compared to indoor HLC. This implies that LTC is a less sensitive means to estimate indoor human biting activities of *An. arabiensis* compared to HLC. Despite lower efficiency, indoor LTC was comparable with that of HLC for *An. arabiensis* in the study area, because there was no significant tendency for the RSE of LTC to be affected by changes in *An. arabiensis* density. This finding was consistent with other studies [7, 13, 15, 18, 35] which support that HLC is the most efficient sampling method for anthropophilic *Anopheles* mosquitoes and for routine monitoring of malaria vectors.

In contrast to these results, several studies [2, 4, 11, 35, 36] showed higher sampling efficiency of LTC compared with HLC for different *Anopheles* species including *An. arabiensis*. This was particularly so in Ahero, Kenya [13] and in Macha, Zambia [27] where *An. arabiensis* was the sole *An. gambiae* sibling species as is the case in the present study. These observed differences in sampling efficiencies could be explained by local variations in host-seeking behaviours of *An. arabiensis* across Africa [37]. Further possible reasons could be attributed to the crude nature of both sampling methods due to lack of operational standard procedures regarding trap placement, operation time, in real world settings [8]. To make more valid comparisons these procedures should be standardized.

Moreover, the high efficiency of HLC versus LTC for sampling host-seeking *An. arabiensis* reflects the basic differences between the two methods. In the case of HLC, a cocktail of stimuli that attract host-seeking mosquitoes such as olfactory, visual cues, volatiles, body heat and humidity are present [38]. Mosquitoes respond to such stimuli and can target the appropriate site for taking a blood meal. By contrast, LTC use mainly visual stimuli. Further, although the presence of human-occupied LLINs besides LTC is expected to augment the trap catches, the excito-repellent effect of the net might have decreased the efficiency of the LTC [39]. However, some studies have shown that using LLINs have little or no impact on the efficiency of LTC [14, 40].

The present results also indicate that the correlation between LTC and HLC for outdoor *An. arabiensis* was statistically significant, but the RSE of LTC was significantly dependent on mosquito density. Such results were expected because *An. arabiensis* can have more diverse alternative hosts outdoors than indoors which might have diverted more host-seeking individuals from outdoor light traps to human and other animal hosts. *An. arabiensis* is known to be flexible in host-preferences and indoor/outdoor feeding based on availability of domestic animals [41, 42]. Furthermore, feeding behaviour of *An. arabiensis* can be influenced by availability of cattle in the homestead [43, 44]. There were plenty of cattle in Edo Kontola and this might be a potential cause for poor performance of the LTC in outdoor situations. Similar to the present results, some previous reports indicate that light traps were less efficient outdoors [45].The present findings, therefore, suggest that light traps may not be a reliable alternative to HLC for sampling *An. arabiensis* outdoors in this setting.

Results also revealed that CB-LTC was less efficient than HLC. It was estimated that on average the RSE of indoor CB-LTC was 0.44 times that of indoor HLC for *An. arabiensis*. The RSE of the trap was not significantly dependent on indoor density of *An. arabiensis*. This finding is in line with a study on *Anopheles aquasalis* in Suriname that showed high efficiency of HLC compared to carbon dioxide baited traps [46]. Yeast-produced CO_2_ was originally developed to be compared with the standard and industrial mosquito attractants specifically CO_2_ from dry ice, pressurized gas cylinders or propane as cheaper and more accessible alternative in remote localities [24]. As a result, most existing evidence show the efficacy of traps baited with yeast generated CO_2_ versus traps baited with the standard attractants [23, 24]. Smallegange et al. [23] reported that traps baited with yeast-produced CO_2_ caught similar number of *An. arabiensis* as traps baited with the standard industrial CO_2_ and addition of human odour increased the trap catches. Based on this, it can be recommended that yeast generated CO_2_ is a promising alternative to HLC and standard mosquito attractants for indoor collection of *An. arabiensis*. Further studies are required to optimize the efficacy of CB-LTC, industrial CO_2_ baited traps and the HLC in Ethiopia and elsewhere.

For the outdoor collection of the same species, there was consistent correlation between CB-LTC and HLC. However, the RSE of CB-LTC was significantly dependent on *An. arabiensis* density. This could be attributed to the diverse alternative hosts that are available in outdoor settings. Further, outdoor environmental factors such as temperature, humidity and wind speed might affect fermentation of yeast-sugar solution and hence the trap efficacy. In addition, persistence, flow rate and impact radius (attractive range) of the CO_2_ volatile have not been optimized and warrant further study.

For *An. pharoensis*, although outdoor LTC captured 0.24 times that of outdoor HLC and indoor CB-LTC caught 0.86 times that of indoor HLC, the RSE of this species in indoor LTC and outdoor CB-LTC were affected by the mosquito density. These collection methods may not be appropriate for estimating reliable *An. pharoensis* human biting rates. This might be attributed to exophilic and zoophilic behaviour of this species [25]. The presence of cattle in the surrounding area might have affected comparison of the sampling methods for this species. Therefore, further studies should consider animal baited traps to assess the impact of cattle on the efficacy of the sampling methods.

However, for indoor and outdoor *An. ziemanni* catches there were significantly consistent relationships between HLC and either of the two methods, respectively. It was estimated that on average, the efficiency of indoor LTC was 0.73 times that of indoor HLC and the corresponding outdoor LTC was 0.39 times that of outdoor HLC. The RSE of LTC was not significantly dependent on either indoor or outdoor *An. ziemanni* density. Based on these results it can be suggested that despite relatively low efficiency of LTC for collecting *An. ziemanni* indoors and outdoors, LTC can be used to determine reliable conversion factors for estimating human biting rates for this species.

Likewise, the relationship between CB-LTC and HLC for indoor and outdoor *An. ziemanni* catches was statistically significant regardless of its density. On average, indoor CB-LTC yielded 0.58 times the number of *An. ziemanni* captured by indoor HLC, whereas outdoor CB-LTC caught 0.36 times the number of the species collected by outdoor HLC. Though *An. ziemanni* is known to feed predominantly on cattle in Ethiopia [31, 47, 48], the present results clearly showed its anthropophilic tendencies as captured by HLC. These contrast some studies [48] which support that CO_2_ attract more zoophilic and opportunistic anopheline species than anthropophilic ones.

For *An. funestus* s.l., there was no consistency between either LTC or CB-LTC compared to HLC indoors and outdoors. The RSE of both methods were significantly dependent on the mosquito density. This means that both methods may not be suitable for collection of host-seeking *An. funestus* s.l. as an alternative to HLC in this area. This is in contrast to some studies [13] that found consistent proportionality between LTC and HLC for *An. funestus s.l*. and the recent analyses [8] that showed that LTC were able to collect similar or proportional number of *An. funestus* s.l. with HLC in Africa. The differences might be attributed to geographical and ecological variations [27] coupled with variations in behaviour of the subspecies in the *An. funestus* group.

Finally, although the main objective of this study was to estimate the RSE of either LTC or CB-LTC against HLC and hence determine conversion factors for effective monitoring of the impact of IRS and LLINs interventions, the RSE of LTC against CB-LTC were also compared. The correlation between LTC and CB-LTC indoors for *An. arabiensis* was weakly positive regardless of mosquito density. In outdoor venues there was no significant correlation for *An. arabiensis* catches using these two methods regardless of mosquito density. Based on these results it is suggested that CB-LTC does not substantially improve sampling of this major vector compared to LTC in this setting for both indoor and outdoor venues.

## Conclusions

Results showed that mosquito collection efficiency of the sampling methods varied by *Anopheles* species. The HLC was more efficient than either of the alternative methods (LTC and CB-LTC) for sampling *An. arabiensis,* the major malaria vector in the study area. However, the RSE of either of the two alternative methods were consistent and comparable with HLC for monitoring *An. arabiensis* indoors, but not outdoors. Therefore, CDC light traps with or without yeast-produced CO_2_ represents an alternative to HLC for large scale indoor *An. arabiensis* surveillance and monitoring because of the various problems associated with using HLC. Adding yeast-produced CO_2_ to light traps does not seem to improve the sampling effectiveness of these traps.

## Abbreviations

CDC: Centers for Disease Control and Prevention
HLC: human landing catch
LTC: light trap catch
CB-LTC: carbon dioxide-baited light trap catch
IRS: indoor residual spraying
LLINs: long-lasting insecticidal nets
RSE: relative sampling efficiency
